# Tuberculous Meningitis Complicated by Communicating Hydrocephalus and Lacunar Infarcts: A Case Report

**DOI:** 10.7759/cureus.88572

**Published:** 2025-07-23

**Authors:** Rony K Varghese, Venkatesh Mittapalli, Govindraju Chikkana

**Affiliations:** 1 General Medicine, Ahalia Group, Abu Dhabi, ARE; 2 Neurology, Ahalia Group, Abu Dhabi, ARE

**Keywords:** complications of tb meningitis, diagnosis of tb meningitis, hydrocephalus, lacunar infarcts, tb meningitis, thwaites criteria

## Abstract

Tuberculous meningitis (TBM) is a life-threatening form of central nervous system tuberculosis (CNS-TB) that often presents with diagnostic and therapeutic challenges, especially in the absence of early microbiological confirmation, and is often associated with complications. We report the case of a previously healthy 32-year-old female who presented with a short history of fever, headache, and altered mental status. Initial cerebrospinal fluid (CSF) analysis revealed a profile consistent with TBM, though microbiological studies were negative. MRI findings showed meningeal enhancement and vasculitic changes. The patient experienced a rapid neurological decline with signs of raised intracranial pressure, necessitating external ventricular drainage and later ventriculoperitoneal (VP) shunting. She also developed hyponatremia likely secondary to syndrome of inappropriate antidiuretic hormone secretion (SIADH), and subsequent imaging revealed multiple lacunar infarcts suggestive of vasculitis-related ischemic injury. CSF culture later confirmed Mycobacterium tuberculosis. The patient responded favorably to empirical anti-tuberculous therapy (ATT), adjunctive corticosteroids, and multidisciplinary supportive care. She made a significant neurological recovery and was discharged ambulant with minimal assistance.

This report underscores the importance of early clinical recognition, prompt empirical therapy, and timely neurosurgical intervention in TBM to reduce morbidity, even when initial laboratory confirmation is lacking. Multidisciplinary involvement, and proactive complication management including that which covers hydrocephalus and hyponatremia, are critical to improving outcomes.

## Introduction

Tuberculosis (TB) is a leading cause of death from an infectious disease globally, with extrapulmonary manifestations occurring in approximately 14% of cases [1]. Central nervous system tuberculosis (CNS-TB), while uncommon, is the most severe form, with a global incidence of around two per 100,000 population. Tuberculous meningitis (TBM), the most frequent and lethal CNS-TB manifestation, is associated with in-hospital mortality exceeding 40% and long-term neurological sequelae in about 28.7% of the survivors [2]. Diagnosing TBM remains a major clinical challenge due to its nonspecific presentation, often mimicking other meningoencephalitides [3,4]. Microbiological confirmation is often delayed or inconclusive due to low sensitivity and limited access to rapid diagnostics [4,5]. Imaging features such as basal meningeal enhancement, infarcts, and hydrocephalus can aid early diagnosis and improve clinical confidence [3]. Delays in initiating treatment have been linked to increased mortality, particularly in resource-limited settings [6].

Complications such as hydrocephalus and cerebral infarcts significantly contribute to poor outcomes in TBM. Hydrocephalus is a common complication of TBM, with communicating hydrocephalus being more prevalent due to either obstruction of cerebrospinal fluid (CSF) flow at the level of the basal cisterns or impaired absorption at the arachnoid granulations [7,8]. Hyponatremia is also seen in 40-50% of TBM cases, often due to syndrome of inappropriate antidiuretic hormone secretion (SIADH) or cerebral salt wasting, and is linked to altered mental status and worse outcomes [9]. We present a rapidly progressive case of TBM in an immunocompetent adult, with multiple complications, successfully managed with cerebrospinal fluid (CSF) diversion and empirical anti-tuberculous therapy (ATT).

## Case presentation

A previously healthy 32-year-old female from Nepal living as an expatriate in the Middle East presented with a two-day history of fever, headache, and irritability. On admission, her vital signs were stable, and neurological examination revealed minimal meningeal signs. Given the short symptom duration and relatively mild presentation, viral meningitis was also considered. Empirical treatment was initiated with intravenous ceftriaxone (2 g every 12 hours), vancomycin (15-20 mg/kg every 8-12 hours), acyclovir (10 mg/kg IV every eight hours), and dexamethasone (8 mg IV every six hours), pending CSF analysis. A lumbar puncture was subsequently performed to guide further management.

**Figure 1:**
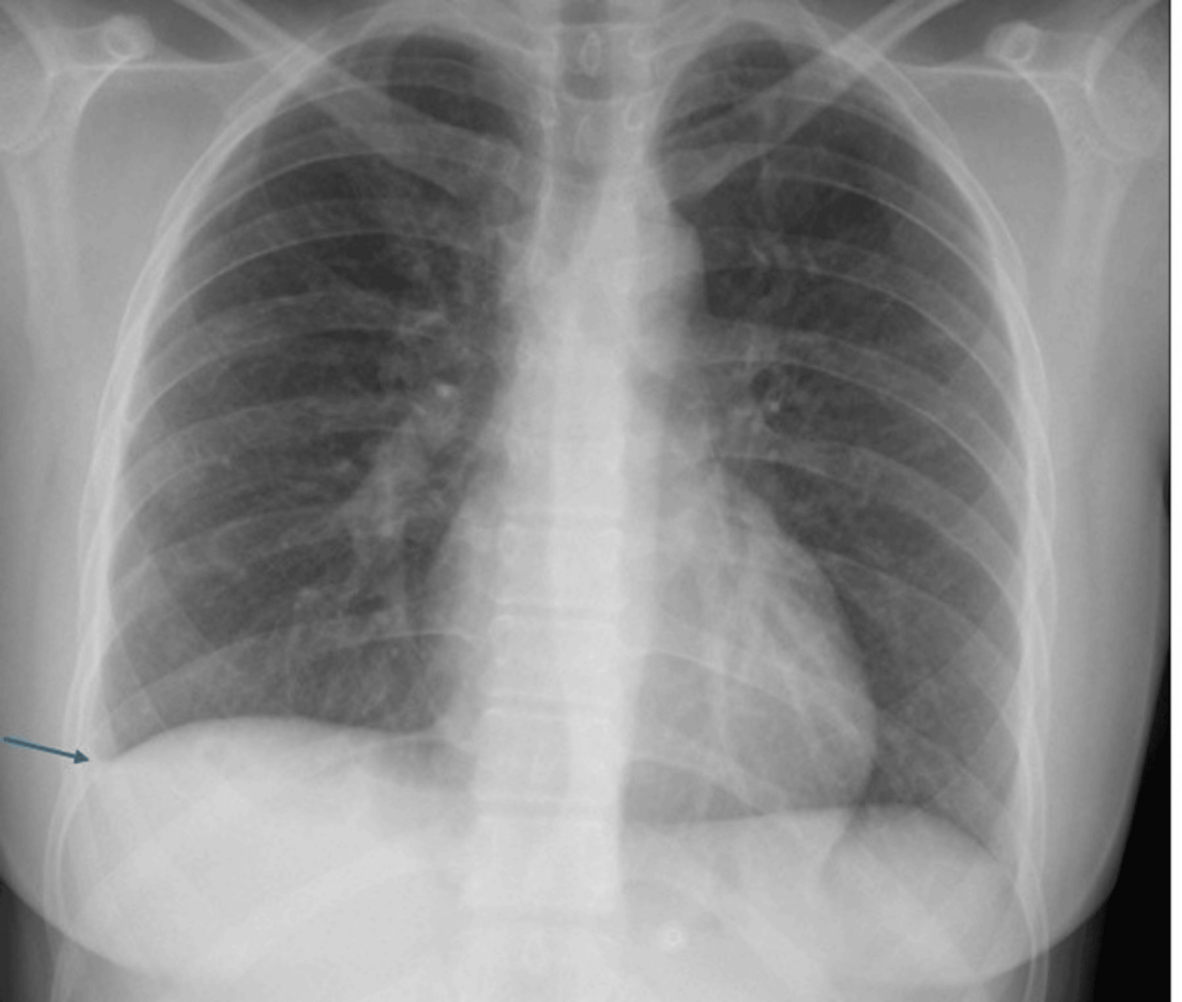
Chest X-ray at presentation demonstrating a mild right-sided pleural effusion The blue arrow indicates blunting of the right costophrenic angle

**Figure 2:**
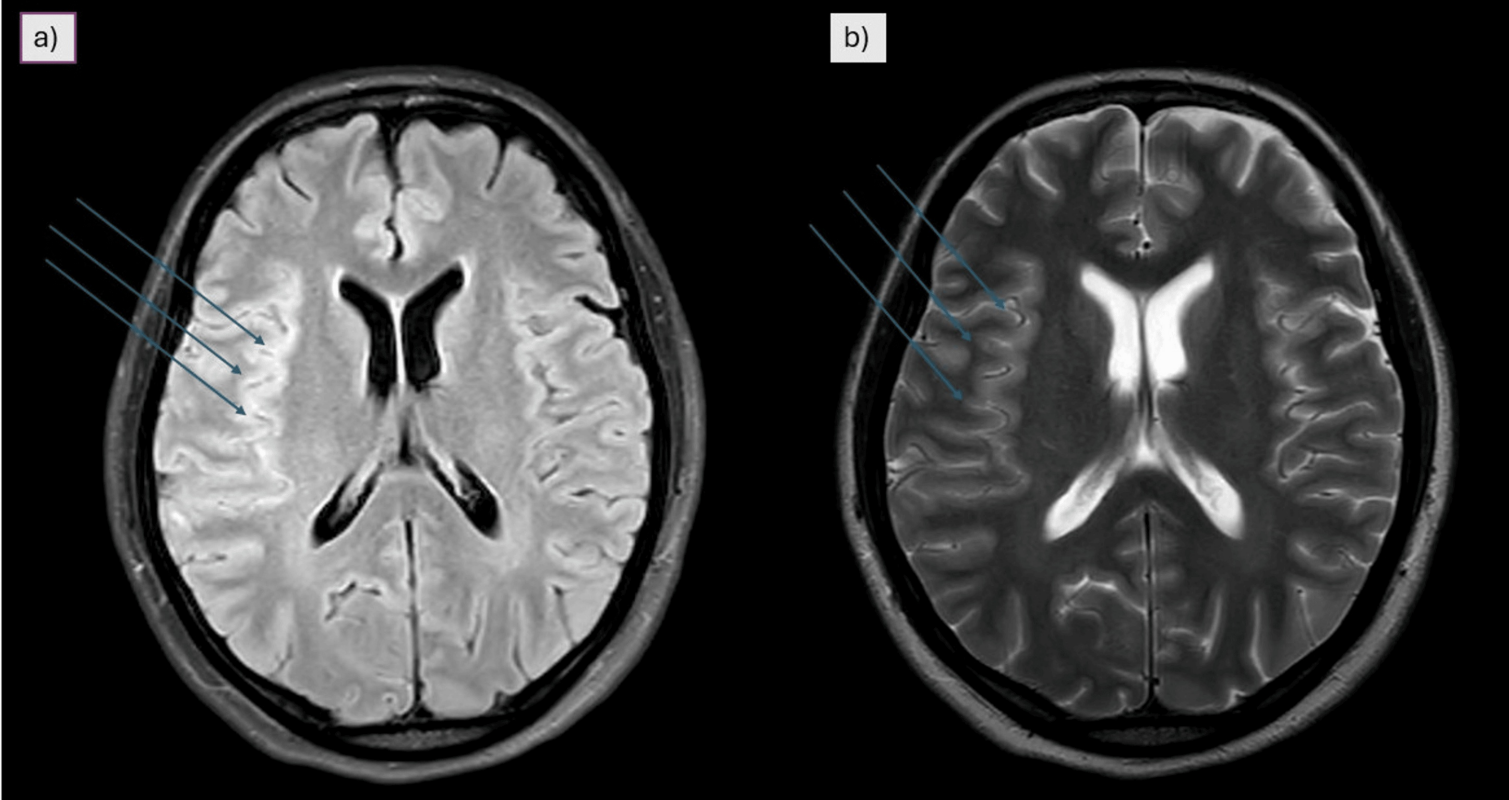
MRI brain (a) FLAIR sequence showing increased gyral thickening and sulcal hyperintensity involving the right temporal lobe and right insular cortex, suggestive of meningoencephalitis. Blue arrows indicate areas of abnormal signal. (b) T2-weighted MRI showing corresponding hyperintensity in the right temporal lobe and insular cortex, consistent with inflammatory changes. Blue arrows highlight the affected regions FLAIR: fluid-attenuated inversion recovery; MRI: magnetic resonance imaging

**Figure 3:**
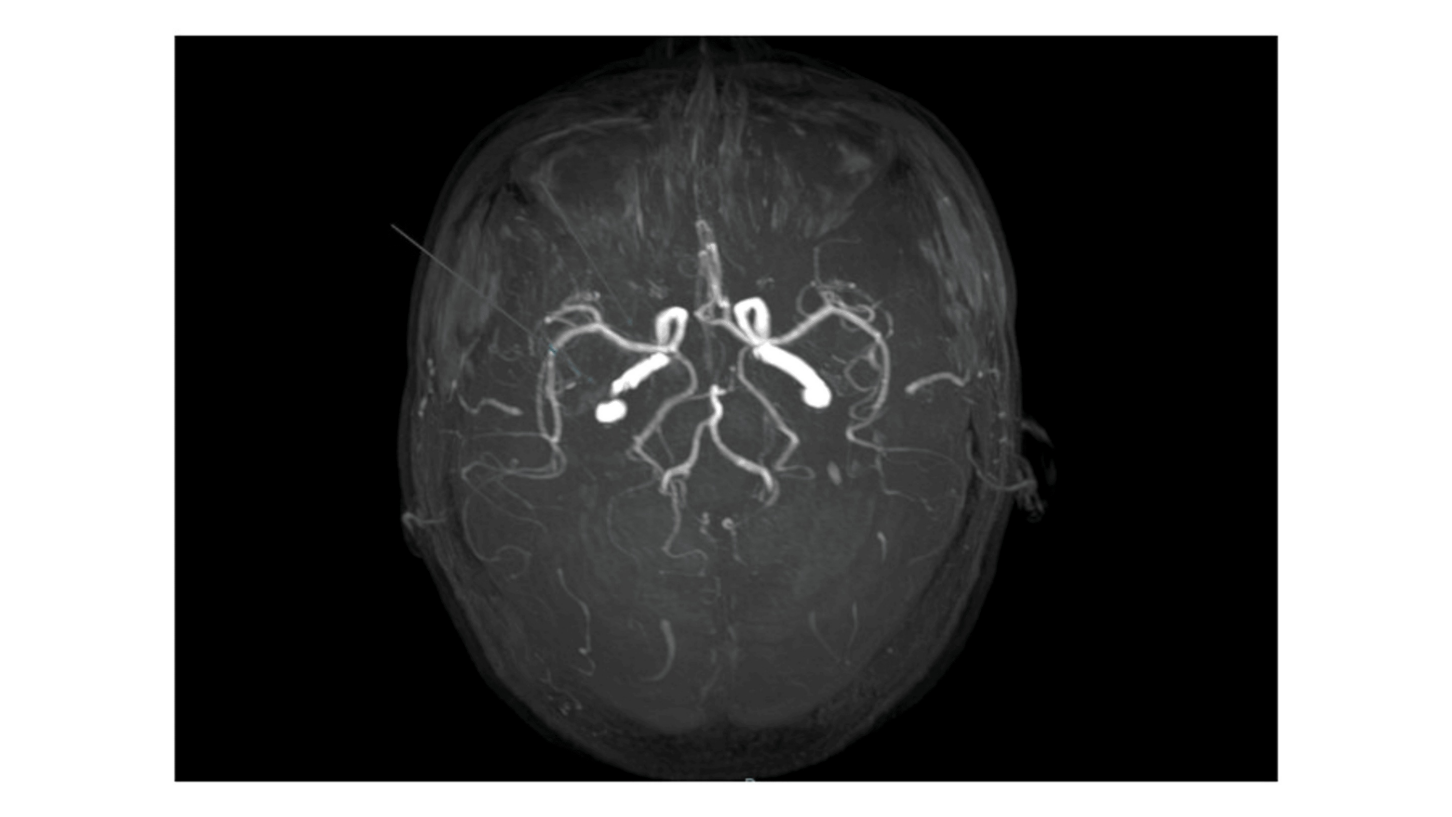
MR angiogram The image shows the irregularity and mild narrowing of the right internal carotid artery and middle cerebral artery

Initial investigations included basic labs, a chest radiograph, which showed mild right-sided pleural thickening (Figure 1). Although a non-contrast CT scan of the brain showed no acute abnormalities, an MRI of the brain with contrast revealed hyperintense signals and contrast enhancement in the right temporal lobe and insular region (Figure 2). Irregularities were also noted in the right internal carotid artery and bilateral middle cerebral arteries, raising suspicion of cerebral vasculitis (Figure 3). CSF analysis demonstrated elevated protein levels (166 mg/dL) and low glucose concentration (26.7 mg/dL), with a lymphocytic predominance (96%). Gram stain, bacterial cultures, and Mycobacterium tuberculosis polymerase chain reaction (MTB-PCR) were all negative.

Although mild clinical improvement was initially observed, the patient subsequently experienced neurological deterioration. Despite the lack of early microbiological confirmation, TBM was suspected based on the CSF profile and radiologic features. Thwaites' diagnostic scoring system [4,10] was applied, yielding a score of 1 (Table 1), strongly supporting a diagnosis of TBM.

**Table 1:** Thwaites diagnostic criteria CSF: cerebrospinal fluid; WBC: white blood cells

Parameter	Patient value	Score
Age	32 years	0
Duration of illness	2 days	+5
Peripheral WBC count	7.05 × 10⁹/L	0
CSF WBC count	145/µL	0
CSF neutrophils (%)	4%	–4

**Figure 4:**
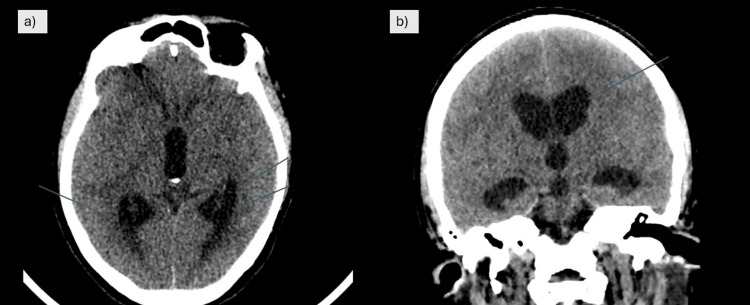
CT brain (a) CT brain (sagittal section) showing prominence of the bilateral lateral ventricles with periventricular hypodensity, suggestive of transependymal CSF seepage. (b) CT brain (coronal section) demonstrating ventriculomegaly CSF: cerebrospinal fluid; CT: computed tomography

On the fifth day of admission, the patient experienced a sudden decline in consciousness, with a drop in Glasgow Coma Scale (GCS) score. A CT scan of the brain revealed effacement of cortical sulci, early ventriculomegaly, and a right temporal hypodensity (Figure 4). She was intubated, and an external ventricular drain (EVD) was placed to manage raised intracranial pressure. Empirical ATT was initiated per regional guidelines. This included isoniazid (5 mg/kg/day, i.e., 300 mg once daily), rifampicin (10 mg/kg/day, i.e., 600 mg once daily), pyrazinamide (25 mg/kg/day, i.e., 1500 mg once daily), and ethambutol (15-20 mg/kg/day, i.e., 1000 mg once daily), alongside ongoing corticosteroids (dexamethasone). As CSF cultures and viral PCR were negative, acyclovir was discontinued, though intravenous antibiotics were continued for a total of 14 days. The patient was subsequently maintained on ATT and a tapering course of corticosteroids.

**Figure 5:**
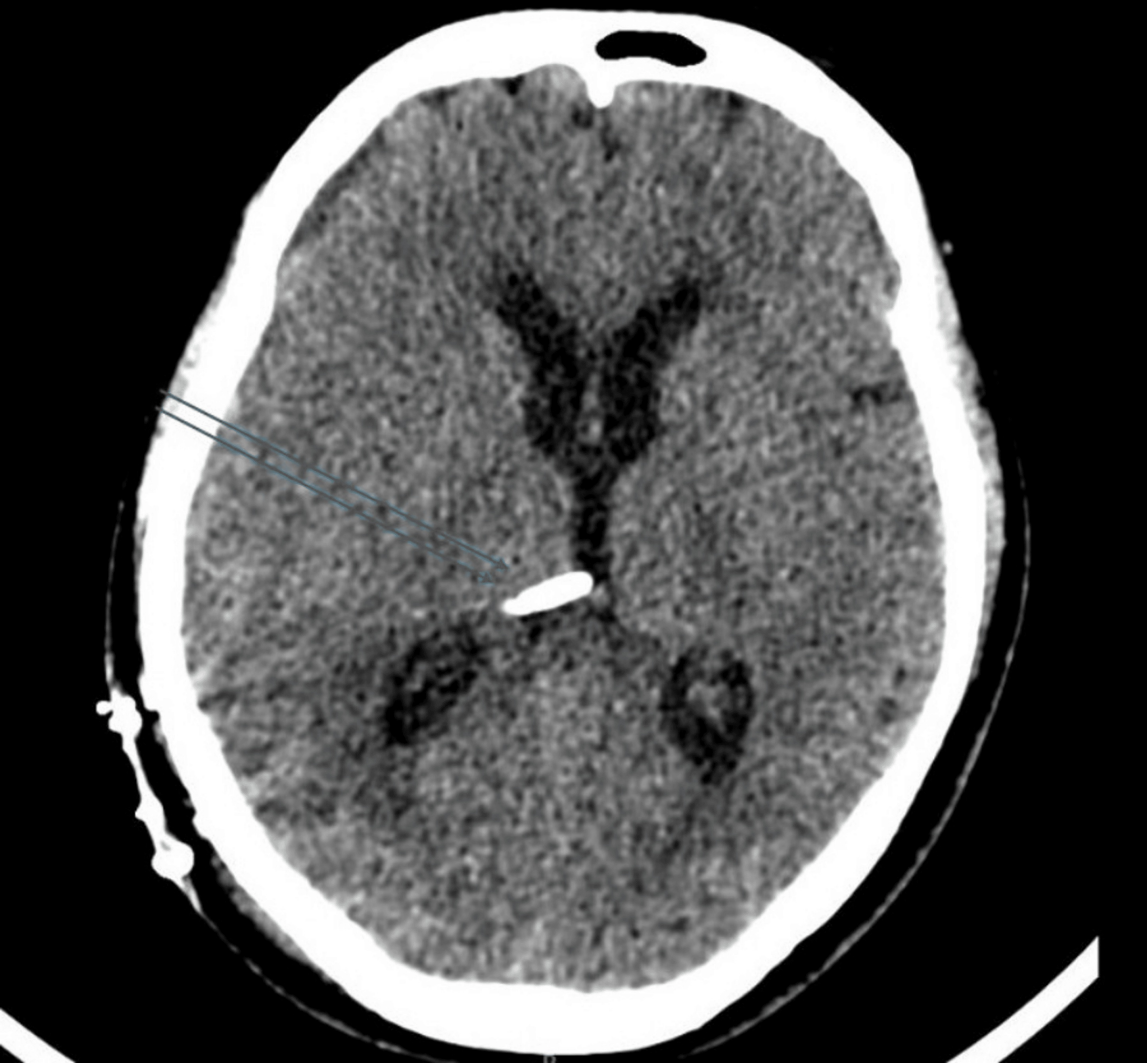
CT brain after EVD placement CT brain following EVD placement showing reduction in ventricular size and decreased periventricular hypodensity, indicating improvement in hydrocephalus. The EVD catheter is visible in the right lateral ventricle, indicated by blue arrows CT: computed tomography; EVD: external ventricular drain

A follow-up CT after the EVD placement showed a mild reduction in ventricular size and improvement in cerebral edema (Figure 5). During the hospital course, the patient developed mild hyponatremia (Table 2), attributed to SIADH. This was managed with fluid restriction, oral salt supplementation, and intermittent administration of 3% hypertonic saline to maintain sodium levels aimed at controlling cerebral edema, resulting in clinical improvement.

**Table 2:** Key laboratory parameters CRP: C-reactive protein; WBC: white blood cells

Parameter	At admission	Day 16	Normal range
CRP, mg/L	1.5	4.0	0.1-5.0
WBC, ×10⁹/L	7.05 (Normal)	7.14 (Normal)	4.5-11
Sodium, mmol/L	130.8 (↓ hyponatremia)	146.7 (Normal)	135.0-145.0
Creatinine, mg/dL	0.67 (Normal)	1.21 (Normal)	0.5-1.20
Hemoglobin, g/dL	10.2 (↓ mild anemia)	9.5 (↓ mild anemia)	11.0-16.8

**Figure 6:**
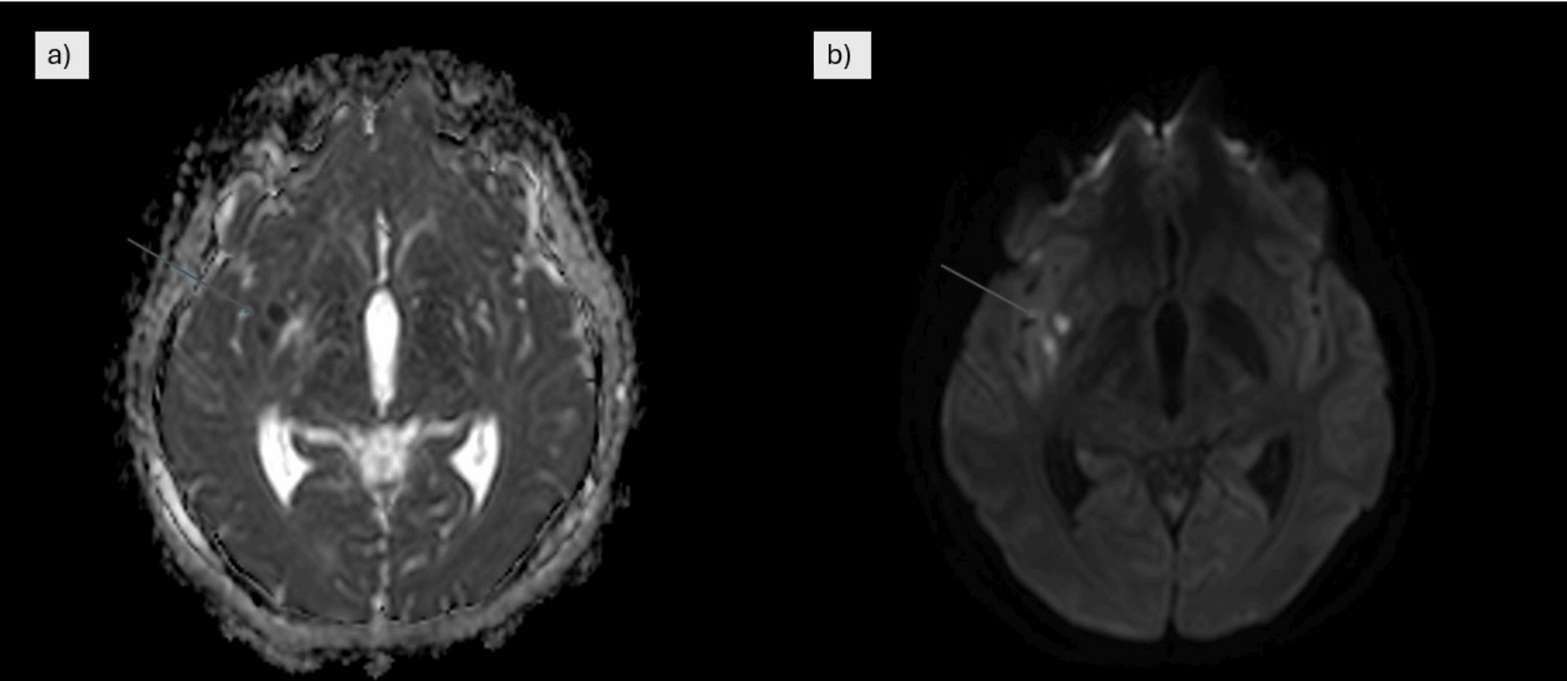
MRI brain (a) MRI ADC map showing acute new lacunar infarcts in the right external capsule and posterior aspect of the right insular cortex, appearing as areas of diffusion restriction. (b) Corresponding diffusion-weighted MRI demonstrating hyperintense acute lacunar infarcts in the right external capsule and posterior right insular region. Blue arrows indicate the regions of infarction ADC: apparent diffusion coefficient; MRI: magnetic resonance imaging

She was extubated on the 11th day following clinical stabilization. However, by the 14th day, the patient developed new-onset mild dysarthria and worsening imbalance, prompting repeat imaging. MRI revealed persistent basal exudates and newly developed lacunar infarcts in the right external capsule and posterior insular cortex, consistent with vasculitic complications of TBM (Figure 6). A repeat lumbar puncture revealed further elevation of CSF protein (213 mg/dL), persistently low glucose (32.1 mg/dL), and lymphocytic predominance (70%). Despite the findings, her clinical condition continued to improve. CSF culture subsequently grew Mycobacterium tuberculosis complex, confirming the diagnosis microbiologically.

The patient was continued on ATT and corticosteroids. Although antiplatelet therapy was considered due to vasculitic infarcts, it was deferred in light of pending neurosurgical intervention. Early involvement of physical medicine and rehabilitation was initiated, and a multidisciplinary team (MDT) discussion led to the decision to pursue definitive neurosurgical management of the hydrocephalus.

Due to persistent communicating hydrocephalus, a ventriculoperitoneal (VP) shunt was placed. Following the procedure, the patient demonstrated progressive neurological recovery. By the time of discharge, she was ambulatory with minimal assistance and had regained normal cognitive function. She was discharged on ATT, with outpatient follow-up arranged in neurology and infectious diseases clinics for continued monitoring and rehabilitation.

## Discussion

This report highlights the diagnostic complexity of TBM in immunocompetent adults. Despite a short symptom duration, the patient exhibited a CSF profile: lymphocytic pleocytosis, elevated protein, and low glucose-alongside MRI findings suggestive of vasculitis and basal exudates. However, initial CSF microbiology, including acid-fast bacilli (AFB) smear and Mycobacterium tuberculosis PCR, was negative, an expected limitation due to the low sensitivity of these tests in TBM. Delays in diagnosis and treatment remain significant contributors to TBM-related mortality. In this context, the diagnostic model proposed by Thwaites et al. (2002) offers clinical value. Their study demonstrated that simple clinical and laboratory parameters could aid in early diagnosis, with an admission scoring system achieving 86% sensitivity and 79% specificity. This tool is particularly applicable in high TB prevalence settings among adults with meningitis and a CSF-to-blood glucose ratio of less than 50%. These findings underscore the importance of integrating clinical, radiological, and laboratory data to guide timely empirical therapy [4,10].

TBM results from hematogenous dissemination of Mycobacterium tuberculosis, with rupture of a subependymal or subpial Rich focus into the subarachnoid space, initiating a cascade of inflammatory responses. This leads to the accumulation of dense basal exudates, obliterative vasculitis of perforating arteries, and obstruction of CSF pathways. These pathophysiological processes underpin the common complications seen in TBM, such as hydrocephalus and cerebral infarction [11]. 

Our patient developed communicating hydrocephalus, a complication more frequently observed in the pediatric population but also reported in up to 65% of adult TBM cases. It is associated with delayed treatment and poorer outcomes if not surgically managed, with CSF diversion often proving to be life-saving in such scenarios [7]. The presence of multiple lacunar infarcts in the external capsule and insular cortex indicated small-vessel vasculitis, which occurs in 15-57% of TBM patients. Cerebral infarctions are often bilateral and localized to deep gray matter regions, referred to as the “tubercular zone". These result from vasculitis and intimal proliferation in perforating arteries, sometimes complicated by thrombosis. Despite the use of corticosteroids, there is currently no established treatment to prevent stroke in TBM. Hyponatremia, a common metabolic complication in TBM, may result from SIADH or cerebral salt wasting. In our patient, serum sodium levels were carefully corrected to a higher target range as part of anti-edema management. [9,12]. 

The patient was promptly initiated on standard ATT along with adjunctive corticosteroids and underwent CSF diversion via an EVD, followed by VP shunting. Early empirical treatment, even in the absence of microbiological confirmation, is strongly supported by international consensus guidelines and clinical evidence, as delay is independently associated with increased mortality. Host-directed therapy with corticosteroids has been shown to improve survival in non-HIV-infected patients and is part of standard care [11,13]. Given the presence of hydrocephalus and infarcts, the expected prognosis would be guarded, with a high risk of long-term neurological sequelae. However, the patient demonstrated significant clinical improvement following CSF diversion and initiation of ATT along with corticosteroids. Nonetheless, long-term follow-up is essential to monitor for cognitive, behavioral, or motor deficits that may evolve over time, as such sequelae are commonly reported in TBM survivors [11].

## Conclusions

TBM poses significant diagnostic and therapeutic challenges, especially in cases where early microbiological confirmation is lacking. This report highlights the importance of integrating clinical findings, CSF analysis, and imaging features to guide early empirical treatment. Prompt initiation of ATT, adjunctive corticosteroids, and timely neurosurgical intervention contributed to a favorable neurological outcome in our patient, despite the presence of complications such as hydrocephalus and cerebral infarction. Early recognition and coordinated multidisciplinary management are essential to improve recovery and reduce the risk of long-term disability in patients with TBM. Continued vigilance is required during follow-up, as late neurological sequelae-such as cognitive impairment, behavioral changes, or motor deficits-may emerge over time. This report underscores the need for heightened clinical suspicion and proactive intervention strategies, particularly in resource-limited settings where delays in diagnosis are common.
